# Supplementary material for: Botulism in Spain: Epidemiology and Outcomes of Antitoxin Treatment, 1997–2019
Source: Toxins (Basel). 2022 Dec 20;15(1):2. doi: 10.3390/toxins15010002 (PMC9863742; doi:10.3390/toxins15010002)
Supplement: Supplementary file 1 [file toxins-15-00002-s001.zip › toxins-2052615-supplementary.pdf]

Supplementary Materials:

Table S1. Characteristics of food-borne botulism outbreaks in Spain 1997–2019, including information on the setting, number of cases involved, detection of the toxin in clinical and food samples, food involved and its trade distribution.

Outbreak	Year	Setting	N of Cases	Positive Result in Clinical Samples (Type)	Food	Distribution	Positive Result on Food (Type)
1	1997	Home	2	Yes (B)	Green peas	Homemade preserved	N/A
2	1998	Home	2	No	Vegetables	Homemade preserved	N/A
3	1998	Home	3	Yes (B)	Cured (Serrano) ham	Homemade preserved	Yes (B)
4	1998	Restaurant	7	Yes	Vegetables	Large-scale food trade	N/A
5	1999	Home	2	No	Asparagus	Homemade preserved	No
6	1999	Home	2	No	Mushrooms	Homemade preserved	N/A
7	2001	Home	2	No	Green beans	Homemade preserved	No
8	2001	Home	4	No	Green beans	Homemade preserved	No
9	2001	Restaurant	2	No	Mixed	Large-scale food trade	N/A
10	2002		2	Yes	Chilli peppers	Homemade preserved	Yes
11	2002		1*	No			N/A
12	2003	Home	2	Yes	Mixed	Homemade preserved	N/A
13	2004	Home	2	No	Green beans	Homemade preserved	No
14	2005	Home	3	Yes	Olives	Homemade preserved	Yes
15	2005		5	Yes	Cured meat	Larg-scale food trade	Yes
16	2005	Home	2	No	Vegetables	Homemade preserved	No
17	2005	Home	4	No	Chilli peppers	Homemade preserved	No
18	2007	Home	2	No	Artichokes	Large-scale food trade	Yes
19	2007	Home	2	No	Vegetables	Homemade preserved	N/A
20	2008	Home	2	Yes	Tuna	Homemade preserved	Yes
21	2009	Home	2	Yes	Vegetables	Large-scale food trade	N/A
22	2009	Canteen	2	No	Chilli peppers	Homemade preserved	Yes
23	2010	Home	4	Yes	Fried tomato	Homemade preserved	N/A
24	2011	Home	2	No	Roasted peppers	Homemade preserved	Yes (A)
25	2011	Home	2	Yes (B)		Homemade preserved	No
26	2011		2	Yes (A)			N/A
27	2011		2	Yes	Paté "micuit"	Homemade preserved	N/A
28	2011	Home	5	Yes (F)	Mixed		N/A
29	2012	Home	2	No	Chilli pepper	Small-scale food trade	No
30	2012	Home	2	Yes (B)	Leek	Homemade preserved	Yes (B)
31	2012	Home	2	No	Pear	Homemade preserved	N/A
32	2013	Home	2	No	Mushrooms	Homemade preserved	Yes
33	2013		2	Yes (B)			N/A
34	2014	Home	2	Yes (B)	Vegetables	Homemade preserved	N/A
35	2014	Home	3	No	Green beans	Homemade preserved	Yes
36	2014	Home	3	No	Beans	Homemade preserved	Yes
37	2016	Home	2	No	Salted and dried roach $^{\Delta}$	Large-scale food trade	Yes (E)
38	2016	Canteen	2	Yes	Beans	Large-scale food trade	Yes
39	2017		2	Yes (B)			N/A
40	2017	Home	2	Yes	Mixed		N/A
41	2018	Home	4	Yes	Beans & carrots	Homemade preserved	Yes
42	2018	Home	2	No	Vegetables	Homemade preserved	N/A

43	2019	Home	4	No	Tuna	Large-scale food trade	Yes
44	2019	Home	7	No	Tuna	Homemade preserved	Yes

N/A: Not analyzed. * Another case was related but symptoms were so soft that hospitalization was not necessary, no samples were taken and no survey was made so it was excluded for the analysis. Δ *C. butyricum* detected in food.

Table S2. Patients with acute adverse events that could be related to botulism antitoxin administration, in Spain 1997–2019.

Year	Sex	Age	Adverse Drug Reaction	Patient's Global Severity
2008	Male	72	Anaphylaxis	Yes
2009	Male	58	Iatrogenic hypotension	No
2013	Male	67	Unknown but reflected in the epidemiological survey	Yes
2013	Female	58	Anaphylaxis and iatrogenic hypotension	Yes

Table S3. Main characteristics related to toxin detected, antitoxin treatment and length of hospitalization in infant botulisms in Spain 1997–2019.

Year	Sex	Age (Months)	Case Classification	Toxin Type †	Antitoxin	Length of Hospitalization (Days)
1997	male	1	Confirmed	B	No	33
1997	female	1	Possible		No	41
1998	male	2	Probable	B (in honey)	No	27
1999	male		Possible		No	
2000	male	2	Confirmed	B	No	39
2007	female	4	Confirmed	Not specified	No	22
2007	female	2	Confirmed	Not specified	Baby-BIG	26
2007	female	1	Confirmed	Not specified	Baby-BIG	33
2008	female	1	Confirmed	Not specified	No	15
2008	female	1	Confirmed	Not specified		23
2009	male	10	Confirmed	Not specified		15
2010	male	7	Confirmed	B		19
2010	male	5	Confirmed	Not specified	No	1
2010	male	1	Confirmed	Not specified		13
2011	female	1	Confirmed	Not specified		19
2011	female	3	Confirmed	B	Baby-BIG	24
2012	female	0	Confirmed	Not specified		33
2016	male	9	Possible		BAT	29
2016	male	5	Confirmed	B		5
2016	male	6	Confirmed	B		15
2017	female	4	Confirmed	B	Baby-BIG	
2017	female	6	Confirmed	B	Baby-BIG	34
2018	male	6	Confirmed	B	No	14
2019	male	3	Confirmed	B	BAT	15
2019	female	1	Confirmed	B	No	10
2019	male	8	Confirmed	B		20
2019	male	2	Confirmed	Not specified	No	13
2019	male	5	Possible		No	13

† Toxin type detection was performed by PCR method and it could not be specified when the only available sample was serum.
